# Acetazolamide Intoxication in an Elderly Patient with Diabetes and Chronic Renal Failure after Cataract Surgery

**DOI:** 10.1155/2020/3764972

**Published:** 2020-01-31

**Authors:** Juliana Maria Kerber, Juliana Dias de Mello, Karolinny Borinelli de Aquino Moura, Gustavo Cardoso da Silva, Iuri Christmann Wawrzeniak, Tatiana Helena Rech

**Affiliations:** ^1^School of Medicine, Universidade Federal do Rio Grande do Sul, Porto Alegre, RS, Brazil; ^2^Intensive Care Unit, Hospital de Clínicas de Porto Alegre, Porto Alegre, RS, Brazil; ^3^Department of Neurology, Hospital de Clínicas de Porto Alegre, Porto Alegre, RS, Brazil; ^4^Graduate Program in Medical Sciences: Endocrinology, Universidade Federal do Rio Grande do Sul, Porto Alegre, RS, Brazil

## Abstract

Carbonic anhydrase inhibitors, such as acetazolamide, are widely used in the treatment of open-angle glaucoma. Severe metabolic acidosis is a rare complication of acetazolamide use, and life-threatening acidosis occurs most commonly in elderly patients, in patients with advanced renal failure, and in patients with diabetes. We describe an unusual case of an elderly patient with diabetic nephropathy and chronic renal failure who presented to the emergency department with severe metabolic acidosis and coma after exposure to high doses of acetazolamide in the postoperative period of ophthalmic surgery. As symptoms of acetazolamide intoxication and uremia are similar, high suspicion is required to detect excessive plasma drug concentrations and intoxication in patients presenting with concomitant uremia. Clinical symptoms are potentially reversible with prompt diagnosis and treatment, including supportive treatment, bicarbonate therapy, and renal replacement therapy. Hemodialysis is particularly helpful in the management of acetazolamide overdose as the medication is dialyzable.

## 1. Background

Acetazolamide is a carbonic anhydrase inhibitor that acts by inhibiting the reversible reaction of hydration of carbon dioxide and dehydration of carbonic acid. This medication decreases the production of aqueous humor within the eye, reducing intraocular pressure [1]. In the kidney, it stimulates the formation of bicarbonate-rich urine in the renal proximal tubular epithelium, producing a diuretic effect and metabolic acidosis [2]. In the central nervous system, it retards abnormal excessive discharge of neurons and lowers intracranial pressure, been widely used for this purpose. This mechanism of action is based on the reduction of cerebrospinal fluid (CSF) secretion by the choroid plexus, where acetazolamide affects both ion transport and protein expression [3]. Therefore, acetazolamide is commonly used to treat elevated intraocular pressure, fluid retention, and epilepsy, as well as other rare diseases such as high altitude sickness, hypokalemic periodic paralysis, hydrocephalus, and Meniere's disease [4].

Serious side effects are rare, but acetazolamide may induce adverse reactions in several organs and systems. In the central nervous system, it causes drowsiness, depression, malaise, fatigue, and paresthesia, while in the kidney, it causes polyuria, metabolic acidosis, and electrolyte imbalance [2, 5]. Advanced renal dysfunction is the most important contraindication to its use, as acetazolamide is eliminated from the body exclusively by the kidneys and its use can induce bicarbonaturia and normal anion gap hyperchloremic metabolic acidosis. Other contraindications include hypersensitivity to the medication, hepatic dysfunction, hypokalemia or hyponatremia, concomitant use of high-dose aspirin, adrenal insufficiency, and hyperchloremic metabolic acidosis [1].

Severe metabolic acidosis is a rare complication, but life-threatening acidosis may occur particularly in elderly patients, in patients with advanced renal failure, in patients with diabetes, and in the presence of concomitant use of nephrotoxic agents. These situations should be seen as risk factors for prescribing acetazolamide.

Altered mental status is common in elderly patients, but the differential diagnosis is not always straightforward because several diseases are possible and may occur concomitantly with polypharmacy [6]. We present here the case of an elderly patient with coma and severe metabolic acidosis with risk factors for acetazolamide intoxication.

## 2. Case Report

A 70-year-old woman with a history of systemic arterial hypertension and type 2 diabetes was admitted to the emergency department with inappetence, altered mental status, hyperreflexia, and myoclonus lasting for 2 weeks. Despite the diabetic retinopathy and nephropathy with advanced renal failure, the patient had been in her usual state until 3 weeks before admission, when she underwent cataract surgery in the right eye at a private clinic, with no intraoperative complications. In the postoperative period, she was maintained on acetazolamide 500 mg 4 times a day for 5 days, and then the dosage was reduced to 250 mg 4 times a day because of incipient neurologic symptoms. Acetazolamide was prescribed for a total of 20 days.

On examination at the emergency department, the temperature was 34.6°C, heart rate 112 beats per minute, blood pressure 98/61 mmHg, respiratory rate 28 breaths per minute, oxygen saturation 95% while breathing room air, and capillary blood glucose 161 mg/dL. Findings from the physical examination were normal, and her pupils were medium sized and reactive to light, with no focal neurologic deficits or signs of meningeal irritation. The urinary output at admission was 2.0 mL/kg/h. Her condition deteriorated with respiratory insufficiency due to a declining mental status, requiring intubation and mechanical ventilation.

Together with the neurologic signs and symptoms, she presented with severe metabolic acidosis. Arterial blood gas analysis obtained during mechanical ventilation with FiO_2_ 0.21 revealed the following values: pH 7.23; PaCO_2_ 26 mmHg; bicarbonate 11 mmol/L; base excess 15 mmol/L; PaO_2_ 118 mmHg; SaO_2_ 96%; anion gap 18 mEq/L; and lactate 0.5 mmol/L. Diagnostic workup did not suggest the presence of an infectious process: C-reactive protein 0.3 mg/L and leukocytes 11.5 × 10^3^/*μ*L. The chest X-ray was normal. A computed tomography scan of the brain was obtained, revealing small old ischemic defects, with no acute abnormal findings. Cerebrospinal fluid analysis revealed clear fluid, with an opening pressure of 20 mmHg, glucose 107 mg/dL, protein 86 mg/dL, and lactate 1 mmol/L.

Eight hours after emergency department admission, the patient was transferred to the intensive care unit (ICU) due to coma and worsening renal function, with a serum creatinine of 4.8 mg/dL (more than twice as much as the value before surgery), urea 275 mg/dL, bicarbonate 11 mEq/L, chloride 106 mEq/L, phosphate 4.3 mg/dL, and anion gap 18.5 mEq/L. The results of blood tests for sodium, potassium, magnesium, calcium, and phosphate were normal (Table 1). Urinary density and sediment were also normal, as were the ultrasound images of the urinary tract. The patient underwent an electroencephalogram, notable for a severe diffuse metabolic encephalopathy, without epileptiform paroxysms. In addition, aggressive intravenous fluids were administered to treat hypovolemic shock, and sodium bicarbonate was infused to reverse severe metabolic acidosis (250 mEq of sodium bicarbonate over 24 hours).

Renal replacement therapy was prescribed because severe metabolic acidosis was resistant to sodium bicarbonate infusion and as the patient remained in coma, probably secondary to uremia exacerbated by the use of acetazolamide. After 2 sessions of intermittent hemodialysis, her neurologic status progressively improved and she was successfully extubated after 3 days in the ICU. On hospital day 6, she was discharged home with a normal neurologic examination and without the need for renal replacement therapy, as serum creatinine levels and urine output returned to baseline.

## 3. Discussion

We described here a case of severe acetazolamide intoxication in an elderly patient with diabetic nephropathy and chronic renal failure. Because acetazolamide is eliminated solely by the kidneys, it accumulates in patients with renal impairment [5, 7]. Altered mental status, ranging from fatigue to deep coma, occurs as a result of the direct effects of acetazolamide on the central nervous system, but hyperammonemia may also contribute to neurologic symptoms during acetazolamide intoxication [8]. Moreover, due to the similarity of symptoms of acetazolamide toxicity and hyperammonemia, drug toxicity may go unnoticed in patients who subsequently develop renal dysfunction and uremia. Unfortunately, ammonia levels and toxicological screen tests were not measured in our patient. However, a classic feature of acetazolamide intoxication, in contrast to uremic symptoms, is the rapid recovery and resolution of the symptoms soon after the start of renal replacement therapy, as the medication is easily dialyzable [4, 9]. This was precisely the clinical course presented by our patient, with rapid clinical and neurologic improvement after only 2 hemodialysis sessions.

The usual dose of acetazolamide in patients with glaucoma ranges from 250 to 1000 mg per day [1]. Our patient received a dose of up to 2000 mg per day in the first 5 days of treatment, which can be interpreted as overdose. Furthermore, some important remarks can be made based on the treatment of this patient: the off-label use of acetazolamide in cataract surgery; the prescription of the medication to a patient with renal dysfunction; and the iatrogenic use of the medication at higher than recommended doses. In addition, advanced age and diabetes are risk factors for acetazolamide toxicity. Taken together, all these clinical features have evolved with the development of acute neurologic impairment and worsening chronic renal disease leading to the severe metabolic acidosis presented by the patient.

The mechanism involved in acetazolamide intoxication is the inhibition of carbonic anhydrase in the renal proximal tubular epithelium, leading to bicarbonate-rich urine and hyperchloremic metabolic acidosis. This mechanism appears to be more pronounced in elderly patients with diabetes or with renal failure [2, 10]. Serum levels of acetazolamide were not measured in our patient, because acetazolamide levels are not commonly measured in clinical settings, which is a limitation of this report. However, the patient's laboratory workup revealed increased acid production, with markedly increased anion gap in the absence of other sources of plasma acidification, such as lactate or ketones. This fact, together with her clinical history of medication overdose, strongly suggests hyperchloremic metabolic acidosis caused by acetazolamide intoxication in combination with high anion gap acidosis due to tubular abnormalities associated with chronic renal failure secondary to diabetic nephropathy. Despite its high intraerythrocytic distribution and plasma binding properties (about 94%), acetazolamide can be effectively removed by dialysis, especially if renal impairment coexists. It is estimated that the rate of acetazolamide clearance in hemodialysis is about 150 mg in 4 hours and the acetazolamide-to-urea nitrogen excretion ratio is 0.16, which allows the prediction of the acetazolamide dialysance in several dialyzing conditions [4]. As expected, the drug was rapidly eliminated in our patient, with rapid and full neurologic recovery.

## 4. Conclusion

Altered mental status is common in elderly patients presenting to the emergency department. However, differential diagnosis is often difficult. The present case provides a rare report of acetazolamide overdose, an uncommon entity that requires high suspicion because of the similarity of symptoms with those of more frequent causes of metabolic encephalopathy. Elderly patients, patients with diabetes, and patients with chronic kidney disease are at the highest risk of acetazolamide intoxication. The deleterious neurologic effects are caused by the direct action of the drug on the central nervous system, together with hyperammonemia and severe metabolic acidosis, since acetazolamide leads to renal excretion of bicarbonate. Renal replacement therapy is recommended early, especially when renal failure is present, leading to rapid resolution of symptoms.

## Figures and Tables

**Table 1:** Laboratory values at baseline (before surgery) and at ICU admission.

Variable	Baseline	ICU admission	Normal range
Sodium (mmol/L)	137	137	135–145
Potassium (mmol/L)	4.9	5.1	3.5–5.5
Calcium (mg/dL)	9.4	8.3	8.5–10.2
Chloride (mEq/L)	—	106	98–107
Phosphate (mg/dL)	4.2	4.3	2.5–4.5
Creatinine (mg/dL)	2.1	4.8	0.5–1.1
Urea (mg/mL)	111	275	16–48
pH	—	7.23	7.35–7.45
Bicarbonate (mmol/L)	—	10.9	22–26
Total CO_2_ (mmol/L)	—	11.8	23–27
Anion gap (mEq/L)	—	18.5	10–20
